# Incrimination of *Aedes aegypti* and *Aedes albopictus* as vectors of dengue virus serotypes 1, 2 and 3 from four states of Northeast India

**DOI:** 10.1099/acmi.0.000101

**Published:** 2020-03-26

**Authors:** Sumi Chetry, Saurav Jyoti Patgiri, Dibya Ranjan Bhattacharyya, Prafulla Dutta, N. Pradeep Kumar

**Affiliations:** ^1^​ ICMR-Regional Medical Research Centre, North East Region, Dibrugarh, Assam, India; ^2^​ ICMR-Vector Control Research Centre, Puducherry, India

**Keywords:** *Aedes albopictus*, Aedes aegypti, Dengue virus, Transovarial transmission, Northeast India

## Abstract

Dengue is an important vector borne disease with a great public health concern worldwide. Northeast India has experienced dengue almost every year for a decade. As studies on dengue vectors from this region are limited, we undertook an investigation to detect natural infection of the dengue virus (DENV) in potential dengue vectors of this region. Adult *Aedes* mosquitoes which were collected were subjected to RT-PCR for detection of infecting dengue serotype. Minimum infection rate was also determined for each positive pool. Out of the total 6229 adult *Aedes* mosquitoes collected, *Aedes aegypti* (63.3 %) was abundant in comparison to *Aedes albopictus* (36.7 %). These specimens (515 mosquito pools) were subjected to RT-PCR for detection of DENV-1, 2, 3 and 4. RT-PCR revealed the existence of DENV in both male as well as female mosquito pools suggesting natural transovarial transmission of DENV in this region. A total of 54 pools tested were positive for DENV-1, 2, 3 serotypes. This study revealed the occurence of DENV in both the potential dengue vectors from this region along with evidence of transovarial transmission which helps in persistence of the virus in nature.

## Introduction

Dengue is an important vector borne disease causing significant morbidity as well as mortality worldwide. A rough estimate of dengue cases occurring every year stands at approximately 390 million and the majority of these are asymptomatic [1]. Dengue is caused by dengue virus (DENV), five serotypes of which are known to cause human infection universally, each antigenically distinct from each other.

Northeast India comprises eight states and except Sikkim, all the other states have reports of DENV activity in the past with the four common serotypes of DENV (DENV-1 to 4) being prevalent in the region [2].

The virus is transmitted by *Aedes* (*Ae*.) mosquitoes, particularly *Ae. aegypti* and *Ae. albopictus* in a human-mosquito-human cycle or by transovarial transmission [3]. Both the vectors are day time feeders. While *Ae. aegypti* breeds mainly in man-made breeding habitats (solid waste), *Ae. albopictus* usually prefers a natural habitat and also breeds with *Ae. aegypti*. Mostly indoor habitat is preferred which is resistant to climatic variations and hence increases the longevity of the mosquitoes [4].

Dengue is widespread in India, especially in the urban environment, with outbreaks being reported previously from many areas [5]. The occurrence of dengue case reports from the northeastern states of India has increased gradually over the years. Probable factors contributing to this could be migration of population from areas where dengue is endemic and increased unplanned urbanization and industrialization which are responsible for creating favourable breeding habitats for the vector mosquitoes. Transovarial transmission (TOT) in dengue is already well established and there are reports from many areas throughout the world [6–8]. Knowledge of transovarial transmission contributing a dengue outbreak is useful in dengue surveillance to explore the possibility of development of models for early warning [3].

Though *Aedes* is prevalent in northeast India, their vectorial status is less documented. Earlier, *Ae. aegypti* was incriminated from Guwahati, Assam for DENV-1 and 2 [9–11]. The role of *Ae. albopictus* in dengue transmission is not known in this region till date. This study was therefore conducted to evaluate the status of the two major dengue vectors in dengue endemic areas of four northeastern states viz. Assam, Arunachal Pradesh, Meghalaya and Nagaland.

## Methods

### Mosquito sample collection and identification

Adult *Aedes* mosquitoes were collected during the period of February, 2018 to February, 2019 in four study sites namely Guwahati (Assam), Pasighat (Arunachal Pradesh), Tura (Meghalaya) and Dimapur (Nagaland). Collection of mosquitoes was conducted in different indoor and outdoor resting habitats using a suction tube. After collection, the mosquitoes were transported to the laboratory, identified to species level using standard identification keys and pooled separately on the basis of study site, day of collection, species, gender and abdominal status (fed and unfed in case of female mosquitoes only) [12].

### RNA extraction

Maximum 20 mosquitoes of same day, location, species, gender and abdominal condition were pooled together. If the number of mosquitoes for one pool exceeded 20, subsequent pools were made with the extra specimens accordingly. Each pool was stored in 50 µl Tri reagent at 4 °C until use. Prior to RNA isolation, the mosquitoes in the pool were thoroughly homogenized till no visible parts remained. After homogenization, RNA isolation was done using Tri reagent as per manufacturer’s protocol [13, 14].

### Reverse transcription polymerase chain reaction (RT-PCR)

The total RNA, extracted from the mosquito pools were initially converted to cDNA followed by PCR for DENV capsid pre-membrane region [15, 16]. The amplified product was again subjected to a hemi-nested PCR, to detect the serotype involved. The presence of individual serotypes in the pools was established by the detection of amplification products having molecular weights 482, 119, 290 and 392 bp for each of the four dengue serotypes (DENV 1–4) respectively using gel electrophoresis.

### Minimum Infection Rate (MIR) calculation

Minimum Infection Rate (MIR) was calculated by assuming that a positive mosquito pool contains a single infected mosquito. MIR was calculated as the ‘ratio of the total number of positive pools to the number of tested mosquitoes, multiplied by 1000’ [17, 18].

### Statistical analysis

Independent samples *t*-test was used for analysing any difference in the means of gender-wise distribution and MIR values of *Aedes* mosquitoes across the four study sites. IBM SPSS Statistics 20.0 was used for all statistical analysis. A *P* value <0.05 was considered as statistically significant.

## Results

### Entomological investigation

A total of 6229 adult *Aedes* mosquitoes were collected from the four study sites. Maximum mosquitoes could be collected from Pasighat, Arunachal Pradesh. The number of mosquitoes collected from Pasighat, Arunachal Pradesh was 2253 (*Ae. aegypti*- 134, *Ae. albopictus*- 2119), Dimapur, Nagaland was 1902 (*Ae. aegypti*- 1858, *Ae. albopictus*- 44), Guwahati, Assam was 1597 (*Ae. aegypti*- 1484, *Ae. albopictus*- 113) and from Tura, Meghalaya, 477 mosquitoes could be collected (*Ae. aegypti*- 470, *Ae. albopictus*- 7). Gender wise distribution of total mosquitoes collected from different locations is given in Table 1. For *Ae. Aegypti,* it was seen that 52.5 % (*n*=2073) of the total mosquitoes collected belonged to the female gender with no significant gender difference in the distribution amongst the four states (*P*=0.869). Majority of the female mosquitoes (90.4 %, *n*=1873) were in the unfed state. For *Ae. Albopictus,* female mosquitoes comprised 79.2 % (*n*=1809) and no significant difference was observed in the gender-wise distribution across the study areas (*P*=0.467). Unfed mosquitoes comprised 95.2 % of the total female mosquito population.

**Table 1.** Species and gender wise distribution of *Aedes* mosquitoes collected from four study sites

Location	*Ae. aegypti*					Total (%)	*Ae. albopictus*					Total (%)	Grand Total
	Male (%)	Female			Two-tailed *p* value		Male (%)	Female			Two-tailed *p* value		
		Total (%)	Fed	Unfed				Total (%)	Fed	Unfed			
Guwahati	662 (44.6)	822 (55.4)	85	737	0.869	**1484** (**92.9**)	52 (46)	61 (54)	3	58	0.467	**113** (**7.1**)	**1597**
Pasighat	64 (47.8)	70 (52.2)	5	65		**134** (**5.9**)	411 (19.4)	1708 (80.6)	83	1625		**2119** (**94.1**)	**2253**
Dimapur	932 (50.2)	926 (49.8)	81	845		**1858** (**97.7**)	7 (15.9)	37 (84.1)	1	36		**44** (**2.3**)	**1902**
Tura	215 (45.7)	255 (54.3)	29	226		**470** (**98.5**)	4 (57.1)	3 (42.9)	0	3		**7** (**1.5**)	**477**
Grand Total	1873	2073	200	1873		**3946** (**63.3**)	474	1809	87	1722		**2283** (**36.7**)	**6229**

**Fig. 1.**
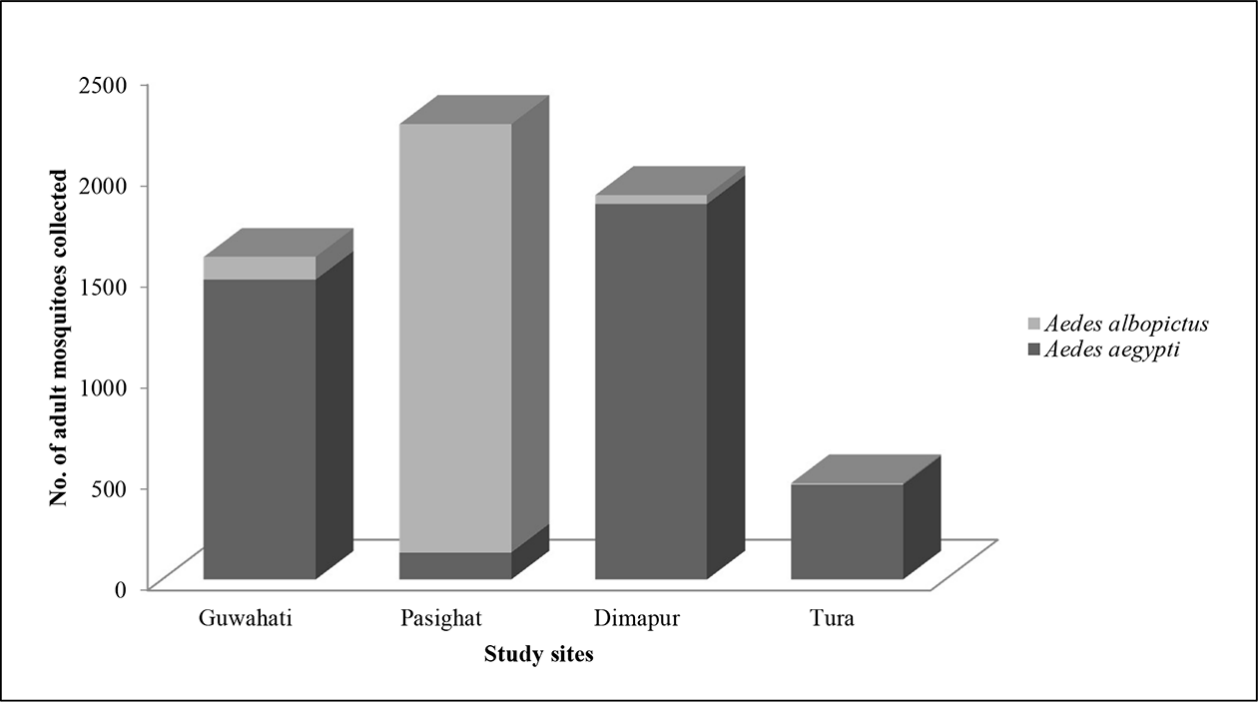
Graphical representation of distribution of *Ae. aegypti* and *Ae. albopictus* in the four study sites.

**Fig. 2.**
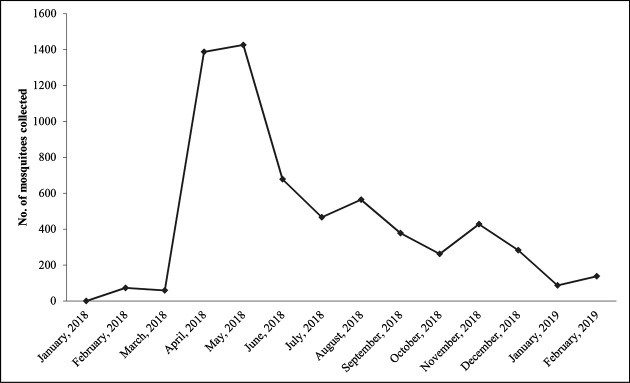
Graphical representation of month-wise *Aedes* collection.

It was observed that distribution of the collected mosquitoes varied in the four study sites of northeast India. *Ae. aegypti* (63.3 %) was observed to be more abundant in comparison to *Ae. albopictus* (36.7 %). In Pasighat, Arunachal Pradesh, *Ae. albopictus* (94.1 %) was predominant as this small township contains and is surrounded by a greater density of natural vegetation as compared to the other three study sites. Again, in Dimapur, Nagaland, *Ae. aegypti* was predominating (97.7 %). Distribution of *Ae. aegypti* and *Ae. albopictus* in the four study areas is shown in Fig. 1. Month-wise collection pattern of *Aedes* mosquito showed that maximum mosquitoes could be collected during pre-monsoon season i.e. April-June, 2018 (Fig. 2).

### Molecular analysis

A total of 515 pools made from the collected mosquitoes were included in the final analysis. During this study DENV-1, 2 and 3 were detected from northeast India (Table 2). Mono-infection with DENV-1, 2 and 3 serotypes was detected in three pools, 46 pools and one pool respectively. DENV-1 and 2 co-infection was observed in three pools, while DENV-2 and 3 co-infection was observed in a single pool (Table 2). Unlike other states, only DENV-2 serotype was detected from Meghalaya.

**Table 2.** State wise distribution of DENV serotypes detected

Location where DENV positivity found	No. of mosquito pools screened for DENV	No. of mosquito pools positive for DENV	Serotypes detected
Dimapur, Nagaland	151	5	DENV-1 (1 pool), DENV-2 (4 pools)
Guwahati, Assam	134	19	DENV-1 (1 pool), DENV-2 (16 pools), DENV-3 (1 pool), DENV-2 and 3 co-infection (1 pool)
Pasighat, Arunachal Pradesh	168	29	DENV-1 (1 pool), DENV-2 (25 pools), DENV-1 and 2 co-infection (3 pools)
Tura, Meghalaya	62	1	DENV-2

### Estimation of Minimum Infection Rate (MIR)

DENV infection was detected in male as well as female mosquito pools. The calculated species and gender wise MIR of each serotype is tabulated below (Table 3). In case of DENV-1, MIR values recorded ranged from 1.2 to 142.9 across the four sites with no significant difference (*P*=0.292) in the values between male and female *Aedes* mosquitoes. In case of DENV-2, MIR values ranging from 1.1 to 49.2 were observed; however, male and female *Aedes* populations in the study sites did not exhibit any significant difference in the MIR values (*P*=0.721). For DENV-3, the MIR of the single positive pool of *Ae. aegypti* (female) collected from Assam was 2.4. Positivity observed in male mosquitoes during the study is a direct indication of natural transovarial transmission (TOT) of the virus in this region.

**Table 3.** State wise minimum infection rate (MIR) of serotypes detected

State	Mosquito species	Gender	Total no. of mosquitoes	No. of positive pools for DENV-1	MIR for DENV-1	Two-tailed *p* value	No. of positive pools for DENV-2	MIR for DENV-2	Two-tailed *p* value	No. of positive pools for DENV-3	MIR for DENV-2
Nagaland	*Ae. aegypti*	Male	932	0	–	0.292	3	3.2	0.721	0	–
		Female	926	0	–		1	1.1		0	–
Assam		Male	662	0	–		7	10.6		0	–
		Female	822	1	1.2		6	7.3		2	2.4
Arunachal Pradesh		Male	64	0	–		3	46.9		0	–
		Female	70	1	14.3		2	28.6		0	–
Meghalaya		Male	215	0	–		0	–		0	–
		Female	255	0	–		1	3.9		0	–
Nagaland	*Ae. albopictus*	Male	7	1	142.9		0	–		0	–
		Female	37	0	–		0	–		0	–
Assam		Male	52	0	–		1	19.2		0	–
		Female	61	0	–		3	49.2		0	–
Arunachal Pradesh		Male	411	1	2.4		9	21.9		0	–
		Female	1708	2	1.2		14	8.2		0	–
Meghalaya		Male	4	0	–		0	–		0	–
		Female	3	0	–		0	–		0	–

MIR: Minimum infection rate.

## Discussion

Dengue has been prevalent in northeast India for at least a decade. Increasing case load with newer geographic invasions every year along with simultaneous circulation of all the four serotypes increases the chances of occurrence of a severe outbreak in near future. *Aedes* vectors are highly prevalent in northeast India. *Ae. aegypti* has already been incriminated as a vector of DENV-1 and 2 from Assam [10, 11]. However, their prevalence and distribution from other northeastern states remains unknown. This is a maiden report which establishes *Ae. albopictus* and *Ae. aegypti* as DENV vectors in these four northeastern states.

DENV-1, 2 and 3 were detected from *Aedes* mosquito populations during the study. No mosquito pool was positive for DENV-4 in the current study. Surveillance in a greater number of mosquito pools over a wider geographical area is required to yield confirmation about the actual status of DENV-4 circulating in the area. Previous studies have also shown that DENV-2 was the predominant serotype in this region and DENV-4, the rarest [2]. In India as a whole, DENV-4 serotype is not very common and from northeast India, previous reports are confined to Arunachal Pradesh and Manipur [2]. Moreover, the serotype replacement phenomenon observed in relation to dengue virus might also be responsible for the absence of DENV-4 serotype in the current study [2]. In Meghalaya, only the DENV-2 serotype was observed, which is in concurrence with the DENV serotype recorded in human cases from the state previously [2].

For DENV-1 and DENV-2, positivity in field collected male mosquitoes suggests natural TOT of *Aedes* mosquitoes of this region. TOT is common in case of *Aedes* and this observation is comparable to earlier studies from this region [3, 10]. Although no significant difference in the MIR values was observed between male and female *Aedes* mosquitoes, the mere detection of DENV in the male population could have an important epidemiological role, as the males have the potential to transmit the DENV infection to females through venereal transmission [19]. During unfavourable seasons, even low MIR might be adequate to maintain the virus in circulation [20].

The presence of multiple serotypes in mosquito pools collected from three of the four states studied implies that the possibility of secondary dengue infections occurring in human subjects residing in the area cannot be ignored. Previous Indian studies from different states have observed secondary infection rates varying from <10 % to as high as >75 % with an overall proportion of 42.9 % (95 % CI, 33.7–52.6) [21]. Considering that secondary infections by a different serotype of DENV can result in increased chances of development of severe forms of dengue such as dengue hemorrhagic fever (DHF) and dengue shock syndrome (DSS), vector control measures become increasingly important to prevent human fatalities [22, 23].

DENV detection in *Aedes* mosquitoes is a routine surveillance practice in different countries including India [24]. For effective epidemiological and clinical management of DENV cases, it is crucial to detect the virus in both field collected *Aedes* mosquitoes and in human samples in a particular area. As treatment for dengue is mainly symptomatic and no effective vaccine is available till date, the only way left to tackle dengue is by vector control and personal protection from mosquito bites. Health officials from a dengue endemic area should educate the community about different measures for mosquito control. Also, routine virological studies should be conducted to have an understanding of the circulating serotypes of DENV.
